# The Development of Altruism with Special Reference to Human Relationships: A 10-Stage Theory

**DOI:** 10.3389/fpubh.2017.00271

**Published:** 2017-10-12

**Authors:** Hing Keung Ma

**Affiliations:** ^1^Centre for Child Development, Department of Education Studies, Hong Kong Baptist University, Kowloon, Hong Kong

**Keywords:** human relationships, altruism, taxonomy, human development, developmental principle

## Abstract

All human relationships involve some form of cost and benefit and altruism forms the foundation upon which human relationships are built. In this paper, a taxonomy of human relationships in terms of altruism was constructed. In the proposed taxonomy, human relationships are categorized into three major groups: primary group, secondary group, and tertiary group. The primary group consists of members that are very closely related to each other either by genetic relatedness (e.g., parents, siblings, and cousins) or social relatedness (e.g., mate and close friends) or both. The secondary group consists of members that are socially related but also less closely related with each other (e.g., people of the same political or religious group, teachers, mentors, acquaintances, neighbors, working colleagues, and strangers). Lastly, the tertiary group consists of members of other species. A 10-stage theory of altruism with special reference to human relationships is proposed. The affective, cognitive, and relationship aspects of each stage are delineated in details. There are two developmental principles of altruism. The first principle states that the development of altruism follows the 10-stage theory and moves from Stage 1: Egoism toward the higher stages of altruism slowly. The second developmental principle states that the taxonomy of human relationships is valid at any stage of altruism development. In other words, people at any stage of altruism are more altruistic toward their kin and mate, and then close friends, extended family members, and so on. They are least altruistic toward enemies and members of non-human species. In summary, the proposed developmental principle of altruism and human relationships is logical and robust. It is formulated based on the major developmental and social psychological theories. The theory has the potential in providing a useful framework for future studies on the development and evolution of human relationships.

Research in the field of social psychology, developmental psychology, evolutionary psychology, and social neuroscience usually did not place emphasis on the relational context. Clark-Polner and Clark (1) argue that “Researchers must consider not only the nature of the actor him or herself, but also the nature of the person with whom he or she is interacting, and crucially, the nature of the existing (or desired) relationship between them” (p. 1). The major objective of this paper is to delineate a full spectrum of human relationships including self, mate, kin, best friends, acquaintances, neighbors, working colleagues, people with similar interests or beliefs, different types of strangers, disliked people or enemies, and other animals. This spectrum of human relationships forms a taxonomy with a hierarchical structure defined in terms of altruism. A 10-stage theory of altruism with a focus on the developmental and relationship aspects is proposed to explain the helping behavior between people of different relationships.

There are several approaches to study the relationship between two persons A and B. Evolutionary psychologists focus on the genetic relation between A and B (2–6), and social psychologists (7–10) study the social factors of human relationships. According to both schools of thought, albeit for different reasons, it is natural that people would act more altruistically to their kin such as parents or siblings than to a common stranger. However, many anthropologists [e.g., Ref. (11–13)] disagree strongly with the genetic claim. Montagu (12) argues while there is indeed a genetic basis to human social behavior, this does not mean that such behavior is genetically determined. He also argues that the behavior of an organism is developed through the interaction between the organism and environment and not between the organism’s genes and environment. “In short, it is the action of the environment upon the organism that influences and makes possible the functional expression of the genes” [(12), p. 11]. As such, the question is: Is the decision of extreme altruism, represented by sacrificing one’s life for other, driven by genetics relatedness or social affection? On the other hand, a number of researchers [e.g., Ref. (14–16)] propose that human social behavior is both biologically and culturally determined. In other words, they believe that the foundation of altruism is both biological (or genetic) evolution and social (or cultural or sociocultural) evolution.

Some psychologists (17–19) use social norms to study the relationships between A and B. Social norms can be defined as “a set of expectations members of a group hold concerning how one ought to behave” [(17), p. 42]. Psychologists and sociologists have attempted to explain prosocial and altruistic behavior by different social norms. Some of the major ones include: (1) Gouldner’s (20) norm of reciprocity, (2) Leeds’ (19) norm of giving, and (3) Berkowitz’s (18) norm of social responsibility. In addition, moral psychologists such as Ma (21–23) use the variable of altruism in his studies of human relationships.

## A Taxonomy of Human Relationships: An Integration of the Biological and Social Factors

### Three Major Classifications of Human Relationships

We propose that there are three major classifications of human relationships: the primary group, secondary group, and tertiary group.

#### Primary Group

The primary group consists of three subgroups: the core group, intimate group, and closely related group. Members of the primary group are closely related either genetically or socially or both. They tend to support each other for group survival and group prosperity. They are most willing to take care of the living and development of fellow members including these members’ survival. They tend to act altruistically toward other members and are willing to sacrifice their belongings or even their life for their fellow members. Some of these altruistic behaviors are innate and some are learned [(24), pp. 68–70 (25)]. The order of importance of these three subgroups to an actor is as follows: the core group, intimate group, and closely related group. In other words, the altruistic orientation of an actor to a member of the core group is the highest, and that to a member of the closely related group is the lowest, with the intimate group in the middle.

##### Core Group

The core group is the most basic and fundamental group of human relationships. It consists of two types of members: genetically related members such as identical twins, parent/child, and siblings (genetic relatedness = 0.5–1.0), and non-genetically related members: spouse and lover who will bear the pro-creational responsibility to produce offspring. In other words, they are either genetically related or have the ability to produce offspring that are genetically related with themselves or with their siblings. They are the main characters of early childhood development.

##### Intimate Group

The intimate group consists of best friends and intimates that are not genetically related. The formation of this group is instrumental and reciprocal, and tends to “solve adaptive problems of survival” [(2), p. 283]. Hruschka (26) argued that “Although subjective closeness is a characteristic of friendship, one can also cultivate such feelings among biological kin. In short, friends and kin are not mutually exclusive categories of relationship. Rather, friendship and kinship are two psychological systems that can exist in hybrid form in the same relationship but have different consequences for behavior” (p. 103). Friendship is perhaps another powerful means for group survival apart from kinship [(2), pp. 277–288 (27)]. Friendship does not appear to be innate and has to be learned and socialized through a systematic pattern of socialization [(26), p. 122].

##### Closely Related Group

The closely related group consists of three subgroups. The terms “coefficient of relationship (*r*)” or “genetic kinship” used by sociobiologists [(28), p. 13 (6), pp. 74–75] are useful for elaboration of this genetic relatedness. Simply speaking, *r* between two persons A and B refers to the proportion of genes in A and B that are identical because of common descent. The *r* between a person (A) and one of his/her parents, son/daughter, or brother/sister in the core group is 1/2 and that between A and one of his/her grandparents, uncle, aunt, nephew, niece, and double first cousin in the closely related group is 1/4. In general, the larger (smaller) the *r* between an actor A and another person (B), the larger (smaller) is the probability that A would carry out an altruistic act for B. This is an extended group of the genetically related core group with lower genetic relatedness. Another two subgroups refer to groups that are basically important social groups that are formed to sustain the transmission of power, wealth, knowledge, and belief which are essential for group survival. These two subgroups are: non-genetically related group I: religious group, political party, interest groups, and non-genetically related group II: superior/subordinate (e.g., teacher/student, mentor/mentee, and leader/follower).

#### Secondary Group

The secondary group consists of three subgroups: mildly related group, stranger group, and antagonist group. Members of the secondary group are less closely related and sometimes are negatively related (i.e., antagonistic to each other). In general, they are less supportive to each other in daily events or in critical events such as matters of survival. Their social behaviors are influenced by social norms, moral values, and traditional doctrines.

##### Mildly Related Group

It consists of acquaintances/common friends (e.g., neighbor, classmate, working colleague, and common friend). This subgroup is an extension of the non-genetically related subgroups in the closely related group. Members of this subgroup are interacting in an instrumental and reciprocal manner. Their valence of interaction is usually light in comparison with that of members in the primary group. In other words, they are less willing to make high cost sacrifices for one another.

##### Stranger Group

It consists of two subgroups. The special stranger group includes elite and disadvantaged or disabled people. The elite (e.g., an outstanding scientist) is someone whom we admire and respect and therefore has a high social status in our perception. The disadvantaged or disabled people are those whom we should give priority during daily interactions (e.g., we should give up our seat in public transport to such people) according to the norm of giving (19). The common stranger group includes people that we are less related to and are thus people we would not regard as necessary to give concession and priority to during daily interaction or critical events.

##### Antagonist Group

It includes your enemies or people you dislike. Naturally speaking, this group of people is less emotionally close with you and may be negative or harmful to you in your daily interactions or critical events. It is the group of people that you would be least altruistic to.

#### Tertiary Group

The tertiary group includes members of a different species and is less related to you either genetically or socially. In general, they are less valued by you in comparison with a human being during critical events of life or death.

## Altruism in Two Hypothetical Moral Dilemmas

To illustrate the study of the human relationships in terms of altruism, let us consider two hypothetical moral dilemmas in Ma’s (21, 29–31) Moral Development Test (MDT).

### The Sinking Boat Dilemma

You and *X* are in a boat which is sinking, but only you or *X* can be rescued. Would you sacrifice yourself so that *X* could be rescued if *X* is … ?

*X_i_*: *X*_1_ = a young stranger, 20 years old; *X*_2_ = an old stranger, 75 years old; *X*_3_ = a famous scientist who is also a Nobel Prize winner; *X*_4_ = your brother or sister; *X*_5_ = your best friend; *X*_6_ = a postman; *X*_7_ = someone you do not like or an enemy; *X*_8_ = a child, 5 years old; *X*_9_ = father or mother.

Now define *A_i_* to be the altruistic orientation of the altruist for *X_i_*, it is hypothesized that

(1) $$(A_{4},\;A_{\text{9}})\text{ > }A_{\text{5}}>(A_{\text{3}},\;A_{\text{8}})>(A_{\text{1}},A_{\text{2}},A_{\text{6}})>A_{\text{7}}.$$

Those *A*-coefficients in a bracket are approximately equal to each other. Equation (1) indicates that the altruistic orientation toward *X*_4_ (sibling) and *X*_9_ (parents) is the highest; and that toward *X*_5_ (best friend) is the second highest; and followed by *X*_3_ (a famous scientist) and *X*_8_ (a child, 5 years old). The altruistic orientation toward other strangers (*X*_1_, *X*_2_, and *X*_6_) is the second lowest and that toward X_7_ (someone you do not like or an enemy) is the lowest. These five categories of people form a hierarchy of human relationships as follows. The *A*-coefficients are given in brackets.

*R*_1_: First kin, close relatives (*A*_4_, *A*_9_).
*R*_2_: Best friends or intimates (*A*_5_).
*R*_3_: Strangers who are very weak, e.g., a blind person; or very young, e.g., a small child of 6 years old; or who are elite of the society, e.g., a famous scientist who is also a Nobel prize winner (*A*_3_, *A*_8_).
*R*_4_: Common strangers (*A*_1_, *A*_2_, *A*_6_).
*R*_5_: Someone you dislike or enemies (*A*_7_).

### Car Accident Dilemma

Suppose 1 day you are on a bus which is in an accident with a car and a heavy lorry carrying dangerous chemicals. Most of the passengers on the bus are injured and it looks as if some might be dead. Fortunately, you are uninjured. You can see flames start to come from under the car and the lorry and you must get away as quickly as possible. However, you feel that you are strong enough to help to move one 20-year-old person to safety, and so you start to drag a stranger near you off the bus. Just as you leave the bus with the stranger, you hear someone (*X*) in the car crying out for help. But you only have time to rescue one person. Would you rescue *X* instead of the stranger from the bus if you recognize *X* as … ?

There are nine *X*s in this dilemma, and they are quite similar to those in the sinking boat dilemma. Eq. 1 also applies for this dilemma.

In the sinking boat dilemma, the major concern of the actor is whether he/she is willing to sacrifice his/her life for the recipient. In the car accident dilemma, the concern of the actor is whether rescuing the person in the car is fair to the person that he/she is rescuing from the bus. Ma (23, 32) has investigated the implications of the actor’s concern in making their moral decision. His empirical findings indicated that the altruistic orientation of an actor is larger to a recipient of close relationship in any situation. In other words, kin altruism is true across different dilemma situations.

## Empirical Studies in the Hierarchy of Human Relationships

Ma (21, 22, 29–31, 33) used several hypothetical moral dilemmas in his MDT to study this hierarchy. In principle, spouse and lover should be in *R*_1_, but for Ma’s previous studies, he focused on first kin (parents and siblings) in *R*_1_, and left spouse and lover for future studies. In the two dilemmas mentioned above, subjects were asked to rate each *X_i_* on a seven-point scale (from *definitely yes* to *definitely no*).

Let us define a coefficient of human relationships $\left(h_{i}^{s}\right)$ as below: $h_{i}^{s}$ = Probability that the actor will carry out an altruistic act to a recipient in *R_i_* category in situation *s*. Situation 1 (*s* = 1) refers to the sinking boat dilemma, where *i* = 1, 2, 3, 4, 5. Similarly situation 2 (*s* = 2) refers to the car accident dilemma in Ma’s (21, 32) MDT.

Let *RB_i_* denote the altruistic orientation to a recipient in *R_i_* category in situation 1 (*s* = 1) (the sinking boat dilemma), where *i* = 1, 2, 3, 4, 5. Similarly *RD_i_* refers to the situation 2 (car accident dilemma). Since the *RB_i_* and *RD_i_* use the seven-point Likert scale, the maximum and minimum values of these scores are 7.0 and 1.0, respectively. The coefficient of human relationship is defined as:

(2a) $$h_{i}^{1}=\;(RB_{i}-\;1)\text{​}/\text{​}(7.0\;-\;1.0)\;=\;(RB_{i}-\;1)\text{​}/\text{​}6.0.$$

Similarly, $h_{i}^{2}$ is defined as:

(2b) $$h_{i}^{2}=\;(RD_{i}-\;1)\text{​}/\text{​}6.0$$

The range of the coefficient of human relationship $h_{i}^{s}$ is from 0.0 to 1.0, where *s* = 1, 2, 3. For example, the coefficient of human relationship $h_{1}^{1}$ is about 0.80, which means that people are very willing to sacrifice their life for their parents or siblings in the sinking boat dilemma. On the other hand, the coefficient $h_{5}^{1}$ for some people is very low, perhaps smaller than 0.25, which means that some people are very unwilling or strongly unwilling to sacrifice their life for a person they dislike in the sinking boat dilemma. Details of the hierarchy of human relationships are given in Ma (32).

## Affective Closeness Hypothesis

### Hypothesis 1

The degree of affective closeness decreases consistently from *R*_1_ to *R*_5_. Correspondingly, the coefficients of human relationship $h_{i}^{s}$ decrease consistently from *i* = 1 to *i* = 5 for a fixed *s*.

The general findings (the mean of the $h_{i}^{s}$ indices) supported the proposed hierarchy of human relationships for both male and female samples (22). In addition, Ma’s (29, 33) findings also supported the proposed hierarchy of human relationships for both English and Chinese samples in different social situations. Apart from a few minor reversals, Ma (23, 31) found that the means of the $h_{i}^{s}$ indices supported the hierarchy among primary school, junior secondary school, senior secondary, and university student samples. In summary, Ma’s findings supported that the degree of affective closeness decreases consistently from *R*_1_ to *R*_5_ in (a) male and female samples, (b) different cultural samples, (c) different educational samples, and (d) different dilemma situations.

## Hierarchical Structure Hypothesis

### Hypothesis 2

The five types of human relationships (*R*_1_–*R*_5_) form a hierarchical structure. Correspondingly, the coefficients of human relationship $h_{i}^{s}$ (for *i* = 1 to *i* = 5, and a fixed *s*) exhibit the following features: (a) the coefficients form a simplex-like correlation pattern. In other words, the correlations of the coefficients in the correlation matrix decrease consistently away from the diagonal in each row or each column from *i* = 1 to *i* = 5. (b) A principal component analysis of the five coefficients will result in two components. The factor plot approaches a concave pattern with the loading on *i* = 3 usually higher than the loading on *i* = 2 or *i* = 4 which are also higher than the loading on *i* = 1 or *i* = 5 in one component. The loadings on the coefficients in other component always decrease or increase consistently from *i* = 1 to *i* = 5.

Ma (33) found that the data in his London and Hong Kong studies supported the hypotheses that (a) the inter-correlations of the five *R_i_* indices displayed a simplex-like structure and (b) loadings of the principal components analysis approximated a two-factor, semicircular configuration with *R* indices ordered by their hierarchical positions. He also found that similar results were found in two different dilemma situations in his Grade 4 to Grade 8 samples in Hong Kong (23).

One major limitation of the above studies is the use of hypothetical moral dilemmas. Gilbert and Ebert’s (34) experimental studies “demonstrate that errors in affective forecasting can lead people to behave in ways that do not optimize their happiness and well-being” (p. 503). Similarly, Pedersen et al. (35) also found in their altruistic punishment experiments that “people inaccurately forecast their affective and behavioral responses to unfairness in experimental games” (p. 7). Based on the affective forecasting studies, some researchers argue that people may not know what they would do when faced with a real-life moral dilemma. On the other hand, Staub and Vollhardt (36) argue that people who have suffered from violence or have experienced natural disasters would become more caring and helpful and they called this phenomenon “altruism born of suffering.” Future studies should focus on people’s information processing and decision making process when they face real-life moral dilemmas. Perhaps field studies in real-life threatening situations may help researchers obtain more useful data on the altruistic behavior of humans. Ethic approval to conduct the field study must be obtained from one’s academic organization’s Institutional Review Board before the study in order to protect the subjects or participants. In some urgent cases such as those unpredictable earthquakes, retrospective ethic approval for experienced researchers may be considered.

Indeed, the use of hypothetical moral dilemmas is a serious limitation. But this is not a good reason to reject all hypothetical dilemma studies before we are able to conduct similar studies in real-life situations. Jones and Rachlin (37) argued that previous studies have found similar results between real and hypothetical situations in social discounting studies and there is no reason to expect different results with social discounting from what they have found even though they are using hypothetical money rewards (p. 285).

## A Proposed Taxonomy of Human Relationships in Terms of Altruism

Composed of the groups mentioned above, the proposed taxonomy of human relationships is given in Table 1. A number of features of this taxonomy should be noted: (1) it does not exhaust all possible types of human relationships, instead it depicts the most standard or common types of human relationships. For example, there are numerous types of genetic-related groups from *r* = 1.00 to almost zero, but we only mention groups with *r* = 1.00, 0.50, and 0.25. For social-related groups, the situation is even more complicated. For example, the common stranger group can be further divided into old stranger, young stranger, child stranger, male stranger, female stranger, stranger from one’s own country, stranger from a foreign country, physically attractive stranger, physically unattractive stranger, and so on. (2) It is better to perceive the choice of these 11 groups to be included here to be typical examples of human relationships. Also, while the choice is not exhaustive, the two hypotheses (1) and (2) mentioned above will be applied to this taxonomy of human relationships. Future studies should be conducted to expand and refine this taxonomy to include other genetically and/or socially related groups. (3) While social norm guides our social interactions with all kinds of people, its influences are more salient in the secondary and tertiary groups. For the primary group, the major factor affecting the social interactions is genetic relatedness and reciprocity.

**Table 1:** A proposed taxonomy of human relationships in terms of altruism.

Classification of relationships	Group	Typical members
**Primary group**		

*Core group*		
*G*_1a_	Genetic-related core group	Parent/child, sibling
*G*_1b_	Non-genetic-related core group	Spouse, lover

*Intimate group*		
*G*_2_	Close friendship	Best friend, intimate

*Closely related group*		
*G*_3a_	Genetic-related group	Uncle, cousin, grandparent/grandson, granddaughter
*G*_3b_	Non-genetic-related group I	Religious group, political party, interest groups
*G*_3c_	Non-genetic-related group II	Superior/subordinate (e.g., teacher/student, leader/follower, head/subordinate)

**Secondary group**		

*Mildly related group*		
*G*_4_	Acquaintance/common friend group	Neighbor, classmate, working colleague, common friend

*Stranger group*		
*G*_5a_	Special stranger group	Elite, idol, army, young child, disadvantaged, or disabled people
*G*_5b_	Common strangers group	People in your society or your country; people from other countries

*Antagonistic group*		
*G*_6_	Antagonist group	Enemies or disliked people

**Tertiary group**		

*G*_7_	Other species	Members of other species

*There are a total of 11 groups of relationships: (a) 6 primary subgroups which include 2 core groups, 1 intimate group, and 3 closely related groups, (b) 4 secondary subgroups which include 1 mildly related group, 2 stranger groups and 1 antagonist group, and (3) 1 tertiary group*.

## Major Features of the Proposed Taxonomy of Human Relationships

### Hypothetical Hierarchical Order of Human Relationships

Based on the above proposed taxonomy of human relationships and our previous studies (21, 22, 29–31, 33), it is hypothesized that:

(3) (H1a, H1b)>H2>(H3a, H3b, H3c)>H4>H5a>H5b>H6>H7

where *H* is the coefficient of human relationships for different groups of people. For example, *H*_1a_ denotes the coefficient of human relationships for people with genetic relatedness of 0.50 (i.e., parents/child and siblings) and 1.00 for identical twins. Those *H*-coefficients in a bracket are approximately equal to each other.

In other words, the altruistic orientation of an altruist to a person of *G_i_* decreases consistently from *H*_1_ to *H*_7_.

Recently, in their study of human relationships in a sample of university students, Ma et al. (38) found that apart from minor reversals in some characters in *G*_3a_ and *G*_5a_, their data in the sinking boat and car accident dilemmas support the following hierarchical order of human relationships:

(4) $$(H_{1a},\;H_{1b})>H_{2}>H_{3a}>H_{5a}>H_{5b}>H_{6}.$$

#### The Relation of the Present Taxonomy of Human Relationships with Current Approaches to Relationship Studies

##### Daphne Bugental

She has proposed five domains of social life and relationships: (1) an attachment domain, (2) a hierarchical power domain, (3) a coalitional group domain, (4) a reciprocity domain, and (5) a mating domain (39). Her theory of five domains of social life and the processes of social interactions in each domain is useful for our construction of the taxonomy of human relationships. For example, the attachment domain is useful for explaining the social interactions and relationships for *G*_1a_ members, the mating domain for *G*_1b_ members, the reciprocity domain for *G*_2_ members, the coalitional group domain for *G*_3b_ members, and the hierarchical power domain for *G*_3c_ members.

##### Margaret S. Clark

She has proposed a theory of communal relationships based on a program of research on giving and receiving benefits in close relationships (40). She also proposes six ways in which relational context varies: relational character, individual differences in approaches to relationships, relationship type, relationship histories and anticipated relationship futures, developmental stage of relationships, and placement of a relationship within wider relationship networks [(1), pp. 2–5]. Among the six ways, the relationship type is more relevant to the present study. In particular, the communal relationships are useful for explaining the relationships in groups *G*_1a_, *G*_1b_, *G*_2_, and *G*_3a_. The communal relationships “provide people with a sense of security and flexibility in seeking as well as giving support. Friendships, romantic relationships, and family relationships (but not always!) exemplify communal relationships” [(1), p. 3].

##### Alan Page Fiske

In formulating a conceptual framework for a unified theory of social relations, Fiske (41) proposed four elementary forms of sociality: (1) in communal sharing (CS) relationship, “the members of a group or dyad treat each other as all the same, focusing on commonalities, and disregarding distinct individual identities” (p. 690). The CS relationships are good for explaining the relationships in groups *G*_1a_, *G*_1b_, *G*_2_, and *G*_3a_. It appears that core family (*G*_1a_, *G*_1b_) and extended family (*G*_3a_) are good examples of CS relationships as members of the group tend to treat others as all the same. (2) “Authority ranking (AR) relationships are based on a model of asymmetry among people who are linearly ordered along some hierarchical social dimension” (p. 691). The AR relationships are good for explaining the relationships in groups *G*_3a_, *G*_3b_, and *G*_3c_. In other words, the AR relationships are common in a big and extended family (*G*_3a_), a political or religious group (*G*_3b_), a business organization (*G*_3c_), and a school (*G*_3c_). (3) “Equality matching (EM) relationships are based on a model of even balance and one-for-one correspondence, as in turn taking, egalitarian distributive justice, in-kind reciprocity, tit-for-tat retaliation, eye-for-an-eye revenge, or compensation by equal replacement” (p. 691). The EM relationships are particularly good for explaining the relationships in *G*_6_, and to a less extent *G*_4_, *G*_5a_, and *G*_5b_. Someone you dislike or an enemy (*G*6) is people you may take an eye-for-an-eye revenge for what they have done to you. (4) Finally, people in a market pricing (MP) relationship “usually reduce all the relevant features and components under consideration to a single value or utility metric that allows the comparison of many qualitatively and quantitatively diverse factors” (p. 692). The MP relationships are in general applicable for explaining the relationships in all groups (*G*_1a_–*G*_7_) in the present taxonomy of human relationships. Future studies should work out how to integrate Fiske’s unified theory with the present taxonomy of human relationships.

##### C. Daniel Batson

Batson et al. (42) found that actors were more willing to help those victims with similar characteristics (personal values and interest) to them than those with dissimilar characteristics whether the difficulty of escape from help is easy or difficult. For dissimilar victims, the proportion of actors that would help in easy escape condition is very low (0.18) and that in the difficult escape condition is quite high (0.64). For similar victims, the proportion of actors that would help in easy and difficult escape conditions is 0.91 and 0.82, respectively (p. 296). In future if experiments are conducted using people of different relationships, some interesting results are expected. For example, in the similar condition, we can choose either kin (*G*_1a_) or best friend (*G*_2_), and in dissimilar condition, we can choose stranger (*G*_5b_) or disliked people (*G*_6_). It would be interesting to see how the different relationships influence the willingness to help in easy and difficult escape conditions.

## The 10 Stages of Altruism and Human Relationships

The development of altruism with special reference to the taxonomy of human relationships over the life span is proposed to follow a 10-stage process. Since the concept of human relationships is defined in terms of altruism, higher stages tend to exhibit more altruistic behaviors to different types of people than lower stages. Three aspects of the stages of altruism are delineated. (i) Affective aspect: the affective aspect is elaborated in terms of the psychological needs (43, 44). According to Maslow’s (44) theory, basic needs are arranged in a hierarchy of prepotency as follows: (1) physiological needs, (2) safety needs, (3) belongingness and love needs, (4) esteem needs, and (5) self-actualization needs. (ii) Cognitive aspect: the cognitive development approach to morality is applied here. In particular, Kohlberg’s (45, 46) theory and Ma’s (30, 43, 47) Chinese perspectives on moral development will be elaborated as far as possible. (iii) Relationship aspect: the relationship aspect of different stages of altruism is explained in terms of the proposed taxonomy of human relationship (21, 30, 32, 43).

From the evolutionary perspective, altruism is defined as “a behavior which is costly to the actor and beneficial to the recipient” and “cost and benefit are defined on the basis of lifetime direct fitness consequences of a behavior” [(48), p. 416]. Ma (32) has also proposed a psychological definition of altruism. There are three criteria of altruism: (1) altruistic behavior must be carried out voluntarily without expectation of a reward. (2) It must aim to benefit the recipient in at least one of the following ways: (a) increases the direct fitness, (b) facilitates the psychological development of higher stages and helps to attain new psychological abilities such as intellectual and social skills, (c) increases the gratification of basic psychological needs such as physiological, safety, belongingness and love, esteem, and self-actualization needs (44), and (d) helps to restore and maintain the emotional stability. (3) Overall, the actor “is doing good” as judged by the recipient [(32), p. 378]. The evolutionary definition focuses on the fitness consequences whereas the psychological definition places emphasis on a wider scope of benefits of the recipient as well as the actor’s motivation of the behavior and the recipient’s perception of the behavior. These two definitions are in some sense complementary to each other.

## Stage 1: Egoism

### Affective Aspect

People at this stage are struggling hard for their physical survival and would try their very best to survive by all means. “They are Machiavellian in maintaining their survival and getting what they want. That is, in order to survive or to get what they want, they would consider to use any means, whether the means are legitimate or not” [(47), p. 177]. The major concern of the actor is his or her self-survival, the survival of other people or other living things are not their concern.

People at this stage do not trust others and have a strong sense of insecurity in the interaction with others. The focus is on the gratification of physiological and safety needs. Psychoanalytically speaking, people at this stage are exhibiting the Id characteristic which is associated with the pleasure principle. Their ego strength is weak, and they tend to seek immediate pleasure and want to satisfy their physiological needs without delay and by all means. According to Krebs and Van Hesteren (49), “the central goals of egocentric accommodation are to relieve tension, to do what one is supposed to, to ingratiate oneself to those in power, and to foster feelings of security” (p. 120). Batson’s (50) concept of social egoism is also relevant here. “We care for others only to the degree that their welfare affects ours” (p. 339).

### Cognitive Aspect

People are thinking with an egocentric perspective. They think that whatever they see, whatever they perceive, and whatever they sense; the other people will see the same, perceive the same, and sense the same. Their logic is simple and straightforward [(43), pp. 7–8]. They would also follow the authority’s command to be altruistic to others just for the sake of avoiding physical punishment from the authority (47). People at this stage act altruistically only to gain approval or to avoid punishment by authorities [(45), p. 17]. They take care of a small group of significant others such as their parents who exert considerable influence on their daily life.

### Relationship Aspect (*G*_0_)

People at this stage are lonely and alienated. Their focus is on the self (*G_0_*) and they do not care of others. They usually do not exhibit kinship and have no friends. People at this stage are dominated by their genetic or biological nature to survive and to generate offspring directly. This is perhaps the initial or primitive stage of altruism and human relationships. The self-survival is basically the major task of everyone at this stage. The concept of group survival, cooperation, and reciprocity is either weak or does not exist at this stage.

## Stage 2: Kin and Mate Altruism

### Affective Aspect

People at this stage tend to trust their kin (parents, son/daughter, or sibling) and spouse only and have a strong sense of insecurity in the interaction with other people except their kin or spouse. The focus is on the gratification of love and belongingness needs.

### Cognitive Aspect

People are thinking with a primary group perspective. They can argue for the benefit of their kin or primary group quite logically and consistently. One of the features of kin and mate altruism is that people would defend their kin for their wrong doings or cover up the kin’s crimes even at the expense of great personal cost.

### Relationship Aspect (*G*_1a_, *G*_1b_)

People at this stage are struggling hard for the survival of their kin and mate and would try their very best to protect them by all means. What is right is to let the kin and mate survive. If they cannot survive, I will not survive too. The primary group at this stage includes mainly the nuclear family (husband, wife, and children). People at this stage build up a wonderful relationship with their kin and mate and form a strong primary group. They exhibit kinship and close relationships with members of their primary group. People would be willing to sacrifice for their kin at a very high cost.

People at this stage are dominated by their genetic nature to survive and to generate offspring. The migration from Stage 1 to Stage 2 is a giant stride in human development. People at Stage 2 shift their focus on self-survival to kinship. The initial and most important step in the development of cooperation, reciprocity, and group survival begins here (4, 5, 51).

## Stage 3: Reciprocal Altruism

### Affective Aspect

People at this stage tend to build up trust and friendship with others. The gratification of love and belongingness needs is usually at high level. They want to find someone to share their happiness as well as grievance. They want to choose someone to lend support to them emotionally and morally. In return, they are also willing to support the counterparts emotionally and morally.

### Cognitive Aspect

People are thinking with a reciprocity perspective. They can argue and calculate for the returning benefits in the interaction with others quite logically and consistently. Equal exchange is their bottom line. It is better to gain more than to lose more in the social interactions. Instrumental cooperation and reciprocal altruism are also found in adults.

### Relationship Aspect (*G*_2_)

People at this stage are struggling hard for self-chosen friendship. Friends support each other in critical situations, and play and enjoy with each other in daily life. Friendship expands their behavioral repertoire to include reciprocal behavior with non-kin. People at this stage build up a positive relationship with non-kin and form a strong bonding with their friends. They exhibit friendship and close relationships with people outside their core family. The formation of friendship is basically social rather than biological. It ties with social and cultural development. The development of reciprocal altruism operates in the formation of intimate friendship.

The migration from Stage 2 to Stage 3 opens a new page in the development of human relationships. It moves from a focus on self-survival and kinship to non-kin reciprocity. It extends the kinship to non-kin relationships.

## Stage 4: Extended Family Altruism

### Affective Aspect

People at this stage would extend their altruistic orientation from nuclear family to a much large group, the extended family, which includes close relatives with genetic relatedness of 0.25, 0.125, or smaller. The gratification of love and belongingness needs is at a high level. The extended family provides a stronger and more secure basis for nurturing their offspring. The love between extended family members appears to be profound, natural, and usually pleasant. Members of the extended family form a powerful primary group which would defend members’ interests, increase their inclusive fitness, and resist any attack from other people or other groups.

### Cognitive Aspect

People are thinking with a primary group perspective. Group discipline and conformity are operated to maintain the solidarity and stability of the group. The good-boy–nice-girl orientation is practiced in daily social interactions (46).

### Relationship Aspect (*G*_3a_)

People at this stage put the group’s interest before their own personal interest. They are willing to sacrifice for the survival of the big family group in a critical situation. People at this stage build up a good relationship with all members of the extended family. They treat members of the group as their close relatives and kin. The concept of kinship is extended to all members of the extended family. Extended family and the nuclear family together form a bigger, stronger, and more effective group in tackling all kinds of survival difficulties and challenges. Mutual altruism or primary group altruism (47) is operated here. The formation of the primary group which include the nuclear and extended family is basically genetic. The extension of nuclear family to big family in the course of development prepares an individual to interact with more different kinds of people (people of same religious or political belief, supervisor and subordinate relationship, acquaintance, working colleagues, strangers from your own society and country, and so on).

The formation of Stage 4 in some sense completes the full structure of kinship. Whereas Stage 2 delineates a simple model of kinship which involves a nuclear family (husband, wife, and their children), Stage 4 describes a complete and full structure of kinship which involves both nuclear and extended families.

## Stage 5: Parochial Altruism

### Affective Aspect

People at this stage would extend their altruistic orientation from kin, intimate, and extended family to two social groups of same religious and political belief, and supervisor/subordinate relationship. The affection between the actor and members of these two groups is moderate to profound, and the gratification of love and belongingness needs is also at a moderate to high level.

### Cognitive Aspect

People of these two social groups work, play, and live together. They think, believe, and cherish similar religious or political or life goal. Similar to the primary groups in Stages 2 and 4, group discipline and conformity are operated to maintain the solidarity and stability of the group.

### Relationship Aspect (*G*_3b_, *G*_3c_)

In parochial altruism, the interacting group consists of (a) political party, religious group, work union, common interest coalition (e.g., football club, chess club, and science society), or a village or a small town where people share similar living styles and values. (b) Supervisor/subordinate (e.g., teacher/student, mentor/mentee, leader/follower, and boss/subordinate). The two groups are in some sense small circle alliance with common interest and common belief. The second group has the function of transmitting skills, values, and knowledge from the older generation to the younger generation. People at this stage build up a good relationship with all members of these groups. They treat members of these two groups as their close friends or even close relatives or kin. In some sense, the primary group is extended to include non-genetically related members. The formation of the small group alliance opens a new page of social and cultural development apart from the formation of intimate or close friends in Stage 3. The in-group bias delineated by Hoffman (52) is clear at this stage. The actor would be altruistic to the in-group members but not to the out-group members. Group conformity favors the solidarity and survival of the small group alliance. People at this stage put the group’s interest before their own personal interest. They are willing to sacrifice for the survival of these two groups in a critical situation.

The first five stages (1–5) of altruism focus on the relationships between an individual and different subgroups of the primary group (self, kin, best friends, extended family, and closely related small group alliance). The driving force underlying the formation of these subgroups for self- or group survival is usually strong (2, 26, 27, 53).

## Stage 6: Social Altruism

In social altruism, the focus is on common friends, neighbors, classmates, and working colleagues. These groups of people form a social network which contributes to the survival of the actor in study, work, business, and daily matters by providing the actor help, cooperation, and collaboration. The formation of this social group widens the scope of social and cultural development.

### Affective Aspect

People at this stage show moderate affection toward members of this social group. The interactions between group members are mainly based on instrumental purposes. People at this stage are only willing to sacrifice for others at a small cost. The affection between the actor and members of this social group is usually moderate, and the gratification of love and belongingness needs is also at a moderate level.

### Cognitive Aspect

People regard this social group as an instrumental basis in daily social interactions. Members of this social group are useful in solving day-to-day problems in study and work.

### Relationship Aspect (*G*_4_)

The development of Stage 6 or above appears to be socially and culturally dependent and less so biologically dependent. The contents of the Stage 6 or above are more related to Pugh’s (54) secondary values which refer to the moral values in a society or in a culture. The major difficulty in the formation of Stage 6 or above is that the emotional closeness between the actor and the recipients in *G*_4_–*G*_7_ is in general much lower than that in *G*_1_–*G*_3_.

## Stage 7: Normative Altruism

### Affective Aspect

People at this stage would consider the gratification of basic needs of the majority of the society in their decision to act in a dilemma situation. They are willing to sacrifice part of their personal interests in order to help those who are deficient of basic needs, in particular deficient of physiological and safety needs.

### Cognitive Aspect

People at this stage are influenced by their school education and religious belief to act according to some normative values such as brotherhood/sisterhood, social responsibility, and moral conscience.

### Relationship Aspect (*G*_5a_)

People at this stage usually act prosocially according to the social norms. In the normative altruism, the focus is on the special stranger group which includes elite and disadvantaged or disabled people. The elite (e.g., an outstanding scientist) is someone whom we admire and respect and therefore has a high social status in our perception. The disadvantaged or disabled people are those whom we should give priority during daily interactions (e.g., we should give up our seat in public transport to such people) according to the norm of giving (19). Normative altruism expects people to be altruistic to the special stranger group which will result in an increase of the survival of this special stranger group. It focuses on behaviors that are prescribed by social norms. The development of Stage 7 is quite dependent on the effectiveness of the social conditioning of the people in a society which is related to the school education, religious belief, and child rearing practice in the society.

## Stage 8: General Altruism

### Affective Aspect

People at this stage usually have a high gratification level of self-esteem needs and will consider the gratification of the basic needs of the majority as more important than the gratification of their own similar basic needs. For example, if both the individual and the majority are suffering from the deficiency of physiological needs, the gratification of the physiological needs of the majority precedes that of the individual.

### Cognitive Aspect

People feel “a sense of obligation to law because of one’s social contract to make and abide by laws for the welfare of all and for the protection of all people’s rights” [(46), p. 175]. In other words, if there is a conflict of interests between the individual and the majority, the individual would act altruistically even at great sacrifices for the majority because of this free agreement and contract.

The reason for an individual to sacrifice himself/herself for the majority is based on an affective self-sacrificing altruistic orientation toward the majority. That is, the small-I should be sacrificed to support the “Big-I” (Chinese proverb). “Small-I” refers to an individual and “big-I” refers to the country or the majority of a group. One of the famous ancient Chinese philosophers, Mo Tzu proposed a doctrine of universal love, which states that “men should actually love the members of other families and states in the same way that they love the members of their own family and state, for all are equally the creatures and people of God” [(55), p. 9]. When asked “what good is such a doctrine,” Mo Tzu answered, “it will bring the greatest benefit to the largest number of people” [(55), p. 10].

### Relationship Aspect (*G*_5b_)

People at this stage focus on the utilitarianism in their behavior. They would act in order to seek the greatest good for the greatest number. Western people tend to put more emphasis on the rational calculation of the utility. On the other hand, Chinese emphasize on the affective self-sacrificial attitude in their behavior in implementing the utilitarian doctrine. It is not only that you must sacrifice for the majority, it is that you should be whole-heartedly willing to sacrifice for the majority because small-I would complete and sustain the big-I.

In general altruism, the focus is on the common strangers especially those in your own country. It is common that people place great emphasis on national survival and patriotism. People at this stage are willing to sacrifice for the survival of their own country at great cost. They would argue based on utilitarianism that people should seek the greatest good for the greatest number in their own country. General altruism strengthens the survival of one’s nation but not the survival of other’s nations. People at this stage decide to help on the basis of the principle of utilitarianism which aims at the greatest happiness of the greatest number. In other words, if there is a conflict of interest between an individual and the majority, the individual should be prepared to sacrifice himself/herself for the majority. Betraying one’s nation is not only immoral but also evil and sinful. The group affection and loyalty (national patriotism) play a critical role in the development of the national group (*G*_5b_) relationships.

## Stage 9: Universal Altruism

### Affective Aspect

People at this stage love all humans including enemies or people whom you dislike. They are willing to perform altruistic acts toward others in a graceful, voluntary, spontaneous, natural, and peaceful manner. The behavior at this stage demonstrates the exemplary characteristics of universal altruism. The ethical principles are based on the concepts of *Jen* (humanity, benevolence, or human heartedness) in Confucianism. *Jen* “has something of the love which parents have naturally for their children. It has something of the compassion which a man of sensitivity feels when seeing an innocent animal slaughtered” [(56), p. 27]. A great Confucian philosopher, Mencius also said, “it is a feeling common to all mankind that they cannot bear to see others suffer” [(56), p. 132].

The features of universal altruism include: (1) everyone has a feeling of distress at the suffering of others. (2) We should love others in the same way we love our own children. (3) A natural way to help others should cause the least disturbance to all parties concerned. (4) The practice of the spirit of “doctor-with-a-parental-heart” means that a doctor should treat all his or her patients as though they are their own children or close relatives. In general, if people in all professions or jobs would hold this spirit of parental heart, then we are sure that we are well treated. (5) If everyone loves others in the same way we love our own children, then it is fair to everyone.

### Cognitive Aspect

People at this stage who are possessors of integrity place great emphasis on their own life style which is closely related to self-actualization (44, 57). According to Krebs and Van Hesteren (49), “Universal altruism stems from a cosmic feeling of oneness with the universe, identification with the species, active compassion for a commonwealth of beings, a full sense of responsibility for the welfare and development of all people, especially the disadvantaged. … The central goal of universal love is to mesh with an ultimately transformed and coordinated nonviolent world” (p. 112). Generally speaking, Krebs and Van Hesteren’s elaboration emphasizes a compassion and responsibility for human. Kohlberg (45) has described a “Stage 7” in his theory of moral development, which is claimed to be a general stage of human development. Kohlberg [(45), p. 344] argues that his Stage 7 is roughly equivalent to Fowler’s (58) sixth stage of faith and that part of the notion of this Stage 7 comes from Erikson’s (59) eighth stage of psycho-social development. Stage 7 is a stage of agape, which “has two essential characteristics: first, it is non-exclusive and can be extended to all, including one’s enemies; second, it is gracious and is extended without regard for merit” [(45), p. 347]. In short, the “Stage 7” person exhibits genuine, self-sacrificing altruistic love to all people. They also perform acts of supererogation that “freely give up claims the actor may in justice demand” [(45), p. 351]. Krebs and Van Hesteren’s elaboration is quite similar to Kohlberg’s Stage 7.

### Relationship Aspect (*G*_6_)

People at this stage act altruistically based on their self-chosen ethical principles. They do not give unconditioned advantage to the majority because of its number of people over an individual as in the case of overall utility, “the greatest good for the greatest number.” Both the individual and the majority in this case are treated as equal and just entities in nature. The altruistic act is also based on a free, natural, autonomous, and good will. In addition, the altruistic act is carried out with the least disturbance to all parties concerned.

In universal altruism, the focus is on all human beings including people from different nations and also people whom you dislike or your enemies. People at this stage demonstrate a broad-minded love to all people. They do not differentiate people according to their nation, gender, age, skin color, religion, and social and political background. They treat all people equal and love them all the same. Universal altruism favors the survival of the whole human species.

## Stage 10: Natural Altruism

### Affective Aspect

According to Dharmasiri (60), “A central theme in Buddhist ethics is that ‘One should treat others in exactly the same way as one treats oneself’” (p. 19). This central theme is also expressed in the concept of benevolence which refers to the deepest love toward other beings and nature. The essential features of benevolence in Buddhism are elaborated by Tachibana [(61), p. 184] as follows:

The chief function of this virtue is to ward off pain and suffering from other beings, whether human or non-human, and further to promote their pleasure and happiness. Its generic maxim, therefore, according to the Buddhist ethical idea, will be: we ought not to hurt mentally and physically our fellow-creatures as well as our fellow-men, but to love and protect them.

In other words, one should be deeply empathetic at others’ distress and suffering. One should therefore try hard to give happiness to others and to relieve others from distress and suffering. As Buddha said, “if I do not go to hell, who else will?” One major difference between the concept of benevolence in Buddhism and the concept of *Jen* in Confucianism is that the former refers to love that embraces all beings or all living things but the latter emphasizes mainly on human beings.

### Cognitive Aspect

Ma (30, 47) argues that people at this stage act altruistically beyond the principles of justice. They give up their own basic just claims, and make sacrifices for other people as well as animals on the basis of good will. The major characteristics of this stage includes: (a) the act is carried out based on good will. (b) The act is carried out naturally, voluntarily, and with a peaceful mind. (c) The actor carries out the act because of a deep and profound feeling of distress at the suffering of the recipient. (d) The recipients may include strangers, enemies, animals, and other living things.

### Relationship Aspect (*G*_7_)

The final stage of altruism put focus not only on human beings but also on other species (30, 47, 61). People at this stage are humane, just, and fully self-actualized. They are regarded as saints rather than common people. They regard all the living things in the universe as equal and would treat them the same in daily and critical situations.

Natural altruism eventually would increase the survival of all species. It can be regarded as gene-cultural co-evolution because it delineates the principle for the survival of human species which is in some sense genetically related as well as the survival of non-human species which are socially related with human beings.

Unless most members of the human species become saint or Buddha, this stage of natural altruism will never be selected by nature. The Stage 10 in some sense describes an ideal ultimate destiny of human development. An interpretation of the characteristics of each stage in hypothetical dilemmas is given in Appendix 1 of the Supplementary Material.

## Differences Between Stages

In order to simplify the discussion, psychological needs in the affective aspect, justice reasoning in the cognitive aspect, and biological and social factors in the relationship aspect will be our focus in the following discussion.

### Affective Aspect: Psychological Needs

#### Physiological and Safety Needs

Since Stage 1 focuses on self-survival, the gratification of the physiological and safety needs becomes its major concern. Psychoanalytically speaking, Stage 1 expresses the feature of Id which is exclusively unconscious and possesses the sex and aggression instincts (43). The selfish and Machiavellian orientation to survive would drive people at this stage to ignore other’s rights to life. When people move from Stage 1 to Stage 2, they start to care the survival of their kin and mate as well as their own. At Stage 3, they also care for their best friends or intimates because of reciprocity. The extended kinship and family in Stage 4 are also valued because it contributes to their inclusive fitness. While a good relationship with people in *G*_3b_–*G*_6_ (and also other animals in *G*_7_) is useful and sometimes important for their well-being, there is no urgency or prepotency in taking care of people in these groups in comparison with those in *G*_1a_, *G*_1b_, *G*_2_, and *G*_3a_. In other words, Stage 5 or above are less related to physical survival needs in comparison with Stages 1–4.

#### Belongingness and Love Needs

Love needs starts at Stage 2 when people at this stage take care of their kin and mate, and extends to Stage 3 for their intimates and Stage 4 for their members of extended family. Belongingness needs also starts at Stage 2 when people build their nuclear family and is extended to Stage 4 for their extended family. At Stage 5 or above, people generalize their love to different kinds of recipients (*G*_3b_–*G*_7_) but usually at a lower gratification level.

#### Esteem Needs

The gratification level of the esteem needs increases from Stage 2 onwards. At Stage 7 and above, the socialization of social norms, national identity, and universal love through education, child rearing practice, and religious activities would help people develop a clear esteem from others as well as their own self-esteem. The reputation and dignity of their national group are sometimes as important as their own reputation and dignity.

#### Self-actualization Needs

The gratification of self-actualization needs is usually low at Stage 7 or below. An obvious increase would be found at Stages 8–10 where people exhibit a broad-minded perspective and universal love in the interaction with others. It is argued that people at Stage 8 or above usually have an altruistic personality (62), a predisposition to help others whether they are closely related with them or not. They are more willing to sacrifice their lives for others in case of emergency. In other words, they should have a weaker tendency to gratify the physiological or safety needs in such emergent situations (43, 62).

#### Conflicts between Two Needs

When there is a conflict between two psychological needs, people at a higher stage would place more emphasis on higher order needs and those at a lower stage would care of their lower order needs. For example, Stage 4 people would be more willing to sacrifice for members of their extended family at high cost than those at Stage 1 or 2. Another example is that people at Stage 7 or above are more willing to help a stranger when he or she is in need than people at Stage 6 or below.

### Cognitive Aspect: Justice Reasoning

#### Pre-Moral Stage

People at Stage 1 basically do not argue or think morally about their behavior. They just follow their life instinct to survive. That is, their major concern is self-survival. Things that help their survival are right and things that do not help or damage their survival are wrong. This is a pre-moral stage. Morality starts at Stage 2 or above when people forms different types of relationships (*G*_1a_–*G*_7_).

#### Reciprocity

It starts from Stage 2 with a biological origin for *G*_1a_ and social origin for *G*_1b_. The biological reciprocity is extended in Stage 4, whereas the social reciprocity is vividly exhibited in Stage 3. Reciprocal altruism at Stage 5 is still quite strong but that at Stage 6 or above tends to decrease significantly.

#### In-Group Bias

Members of a certain group are usually altruistic to their group members only. In other words, people in Group A would be altruistic to Group A member but less so to members of other groups. This in-group bias altruistic tendency starts at Stage 2 (*G*_1a_ and *G*_1b_) and appears to be strong at Stages 3–5 which involve different types of primary groups (*G*_2_–*G*_3c_). In addition, this tendency also accounts for Stage 6 which consists of a secondary group (*G*_4_).

#### Norm-Abiding

The effect of social law and social norm on one’s altruistic behavior becomes much salient at Stage 7. On the other hand, social norm exists as early as Stage 2 (e.g., Norm of Filial Piety). But at lower stages, the other factor such as genetic or intimacy factor is more dominating. The effect of social norm decreases at Stage 8 or above where utilitarianism and universal love are more influential.

#### Utilitarianism

Utilitarian perspective is the major theme of Stages 8 and 9. On the other hand, the golden doctrine, “to seek the greatest good for the greatest number” can be applied to resolve conflicts in various groups of relationships in Stages 2–6 or 7.

#### Justice and Humanity

The self-chosen ethical principle and humanity in Stage 9 and the Sainted altruism in Stage 10 denote the ultimate stage of altruism development. These concepts are less emphasized in lower stages.

### Relationship Aspect

#### Group Survival

If everyone strives to survive and ignores other’s survival, everyone still contributes to the survival of the group. But this is not the best way for the group to survive. The formation of different groups of relationships (*G*_1a_–*G*_7_) in principle increases the survival of every member of a group and thus increases the survival probability of the whole group. It is argued that each stage of altruism contributes significantly to the survival of the whole group provided most of the members of each stage practice the stage contents quite fully or adequately. Since each stage contains the major features of the preceding stages, the higher the stage it is, the more powerful it contributes to the survival of the group.

#### Emotional Closeness

The emotional closeness with others at Stage 1 is the lowest. A person at Stage 1 loves himself or herself but does not love any other people. People at Stage 2 love their kin and mate and people at Stage 3 love their best friends and so on. Finally, people at Stage 10 not only love humans but also other animals and living things. The scope of emotional closeness tends to become broader and perhaps also more profound to all the others through development over time.

The 10-stage theory of altruism and human relationships is outlined here in Table 2. Each stage is related to the human relationships in a particular group (*G*_1_–*G*_7_).

**Table 2:** A 10-stage developmental theory of altruism with special reference to human relationships.

Stage	Altruism	Group classification	Human relationships
1	Egoism	*G*_0_	Self/individual
2	Kin and mate altruism	*G*_1a_, *G*_1b_	Kin and mate
3	Reciprocal altruism	*G*_2_	Intimate
4	Extended family altruism	*G*_3a_	Extended family
5	Parochial altruism	*G*_3b_, *G*_3c_	Common interest group
6	Social altruism	*G*_4_	Social group
7	Normative altruism	*G*_5a_	Special ability group
8	General altruism	*G*_5b_	Nation and common strangers
9	Universal altruism	*G*_6_	Human species
10	Natural altruism	*G*_7_	Non-human species

### Two Developmental Principles of Altruism

There are two developmental principles of altruism. The First Developmental Principle states that the development of altruism follows a ten-stage process from egoism to natural altruism. The scope of social interactions becomes broader and the degree of altruistic orientation becomes stronger and more profound towards different types of people (*G*_1_–*G*_6_) as well as members of different species (*G*_7_) in a slow process over time. The Second Developmental Principle of Altruism states that the taxonomy of human relationships is in general valid at any stage of altruism development. In other words, people at any stage of altruism are more altruistic toward their kin and mate, and then close friends, extended family members, and so on. They are least altruistic toward enemies and members of non-human species. For people at higher stages such as Stage 9 or Stage 10, their H-coefficients (*H*_1_–*H*_7_) would be very high and usually approach 1.00. They are highly altruistic toward all other people (*G*_1_–*G*_6_) and members of different species (*G*_7_). The details of these two developmental principles should be worked out in future studies. In addition, these two principles should also be empirically tested.

## Limitations of the Theory

(1) *The taxonomy of human relationships*: The proposed taxonomy consists of 10 groups of people and 1 group of members of other species. It is not exhaustive and it may not cover the whole spectrum of human relationships. For example, people with genetic relatedness of 0.125 or smaller may form another genetic-related group. The mildly related group (*G*_4_) and the stranger groups (*G*_5a_, *G*_5b_) may also be extended to include more members or subdivided into more subgroups in future studies.
(2) *The 10 stages of altruism*: It is argued in this paper that the first six stages tend to be cultural universal but significant cultural differences may be found in higher stages (e.g., Stage 7 or above). Future studies should provide different cultural perspectives on the higher stages and also empirical data from different cultures to test this hypothesis. In addition, a much more detailed elaboration on the differences among stages is useful. For example, how biological (genetic) and cultural factors interact to influence the development of each stage of altruism?

## Suggestions for Future Studies

(1) *Personality and altruism*: There is an interesting and challenging topic to discuss. Rushton (62) and Rushton et al. (63) proposed that there is a general trait called altruistic personality. They found that self-report altruism rating correlated positively with peer-ratings of altruism, completing an organ-donor card, and prosocial orientation. In other words, people with an altruistic personality tend to exhibit more prosocial and helping behavior than the other people. Ma et al. (64) also found that prosocial orientation was associated negatively with psychoticism and neuroticism. In general, altruism tends to be associated positively with personality such as altruistic personality and negatively with unhealthy personality such as psychoticism and neuroticism. It is argued that higher stages (Stage 8 or above) of altruism are associated positively with healthy personality such as altruistic personality, moral characters [e.g., humanity, honesty, courage, and responsibility. See Ref. (65)] and negatively with unhealthy personality such as psychoticism and neuroticism. Future studies should focus on the study of the underlying personality traits in each stage.
(2) *Moving forward and backward among stages*: The moving forward and backward among stages depends on a number of factors. Some major ones include: (1) maturation and biological factors (as one grows, one tends to develop higher stages), (2) the affective interaction between the actor and the recipients (the degree of affective orientation toward the recipients may have an effect on the moving forward or backward; you love more people, you will move forward; but if you hate more people, you will move backward), (3) the personality of the actor (people who develop psychotic or neurotic personality tend to move backward among stages; while people with high moral identity tend to move forward), (4) the cognitive development of the actor (high level of cognitive development favors moving forward), and (5) the situational factors (people in a negative and fierce environment tend to move backward). This is an important issue for researchers to work on in their future studies. It is beyond the scope of this paper to deal with this issue adequately here.
(3) *An integrated approach to human relationships*: A discussion on the relation of the present taxonomy with the contemporary approaches to relationship studies is given on pp. 14–15. Some suggestions for future studies have also been presented. It would be interesting to integrate different approaches to relationships into one single theoretical model.
(4) *Empirical test of the 10-stage theory of altruism*: Future studies may include: (a) are the 10 stages form an invariant sequence of development? This may require long-term longitudinal studies to test this hypothesis. (b) Are there gender differences in these stages? For example, do male people tend to use the higher stages than the female counterparts (or vice versa) in solving the moral dilemmas? (c) Are there cultural differences in these 10 stages of altruism? (d) Do the stages of altruism predict altruistic behavior? (e) What are the practical use of the 10-stage theory of altruism in school education, child rearing practice, and medical field?

## Conclusive Remarks

In this paper, a taxonomy of human relationships in terms of altruism was constructed to explore how both genetic and social factors influence altruistic behavior to produce human relationships of varying emotional closeness. A 10-stage theory of altruism with special reference to human relationships is also proposed. In comparison with the author’s previous studies and the existing approaches, the present theory has achieved the following objectives: (1) previous hierarchies of human relationships include five categories (*R*_1_–*R*_5_) only (21, 22, 29, 32, 33). The present taxonomy deals with 11 categories of relationships (*G*_1a_–*G*_7_). In other words, the present theory deals with a substantial part of the full spectrum of human relationships. (2) Previous theories of altruism have five to seven stages (30, 43, 47, 49). The present theory includes all the major features of the previous theories. It has 10 stages with a focus on affective, cognitive, and relationship aspects. The relationship aspect is new in the study of the development of altruism. (3) The stage of altruism is delineated in parallel to the taxonomy of human relationships. Each stage of altruism has a focus on one or more relationship groups.

In summary, the proposed developmental principle of altruism and human relationships is logical and robust. It is formulated based on the major developmental, social psychological, and human evolution theories. The theory was also constructed with reference to the empirical findings in the psychological studies of the human relationships (21–23, 31, 33, 66). The theory has the potential in providing a useful framework for future studies on the development and evolution of human relationships.

## Author Contributions

The author confirms being the sole contributor of this work and approved it for publication.

## Conflict of Interest Statement

The author declares that the research was conducted in the absence of any commercial or financial relationships that could be construed as a potential conflict of interest.
