# Supplementary material for: The Development of Altruism with Special Reference to Human Relationships: A 10-Stage Theory
Source: Front Public Health. 2017 Oct 12;5:271. doi: 10.3389/fpubh.2017.00271 (PMC5649141; doi:10.3389/fpubh.2017.00271)
Supplement: Supplementary file 1 [file presentation_1.pdf]

Appendix 1: INTERPRETATION OF THE STAGE CHARACTERISTICS IN HYPOTHETICAL DILEMMAS

In order to delineate the characteristics of each stage more clearly, the author has constructed the following responses to some hypothetical moral dilemmas based on the above theory as well as his previous studies (Ma, 1988, 1992b, 1997, 2013). The responses focus on the relationship aspect of each stage and less so on the other two aspects, affective and cognitive. Chinese proverbs are presented with quotation marks.

STAGE 1: EGOISM

"If I don't care for myself, Heaven and Earth will be destroyed". My survival is more important than anything. In any case, everyone should try to survive first, the others including my spouse and children are not my major concern.

STAGE 2: KIN AND MATE ALTRUISM

I love my wife and children. What is the meaning of my life if I can't protect them and save their life in a critical situation. If I die for them; my spirit, my thinking, my ideology, and my inspiration will still be with them; and they will try to realize all the things that I don't have time to finish. I regard myself and my kin and mate as a single whole, some old members will die and some new members will be born, but the single whole will last and persist for ever.

STAGE 3: RECIPROCAL ALTRUISM

"Life will be no regret if you can have one or two intimates that understand you, love you, and accommodate you". I am willing to sacrifice for my best friends in a dilemma situation because I know that my best friends will also do the same to me. Profound friendship does not necessary build on instrumental purpose, it can build on selflessness, love, and trust. Best friends not only help me in many ways but also help me to survive happily.

STAGE 4: EXTENDED FAMILY ALTRUISM

I love my big family because it is strong and helpful. Blood is thicker than water. Members of the big family have the same root and the same ancestors. If we unite, we will be stronger; otherwise we will be defeated by others.

STAGE 5: PAROCHIAL ALTRUISM

I am willing to sacrifice a lot of my money, my time, and my effort for my friends in my church (or my fellow comrades of the same political party) because we have the same belief and value. All of us are loyal to our religious group and political party. The survival of our religious group (or political party) is more important than our individual survival. I only care of my own religious group (or political party), others' groups are not my concern.

I love my teacher, my mentor for his or her teaching, support, inspiration, and encouragement that shape my intelligence, morality, and character. "A day as my teacher will be my father for thousand days".

STAGE 6: SOCIAL ALTRUISM

I help my neighbors who will help me in return when needed. "A relative far way is less helpful than a nearby neighbor". Acquaintance and working colleagues should mutually help and support each other. My sacrifice for my neighbors, acquaintance, and working colleagues is usually moderate because we are less intimate in comparison to my best friends, my teachers, and my friends in my church or in my political party.

STAGE 7: NORMATIVE ALTRUISM

I am particularly interested in helping the old, the very young (e.g., a child), and the disadvantaged (e.g., a physically handicapped person) because they are less strong and more vulnerable to external disturbances. I hope that "I can respect other's elderly as much as I respect my own elderly, I can love other's young children as much as I love my own children". I am also willing to sacrifice more to those who have contributed significantly to the society (e.g., a scientist) than to other common people to show my respect to these respectable people.

STAGE 8: GENERAL ALTRUISM

If I can only rescue one group among several groups of people, I would choose the Chinese group. I love my country and I would be more willing to sacrifice for my country than for other countries. "The rise and fall of a country is the responsibility of

every man". I can't see my country be defeated by another country. I love all Chinese and want to see them live happily. I am a small-i. I am willing to sacrifice my life without regret for protecting my country (Big-I). After all, "small-i should be willing to sacrifice in order to support the survival and complete the integrated whole of the Big-I".

STAGE 9: UNIVERSAL ALTRUISM

I love all people including my enemies or people whom I don't like. Chinese people, African people, American people and people from different countries appears to me the same. They are all equal. I love them as much as I love the Chinese people. I can't bear to see people in distress and seeking help in vain. I will definitely give my hand even though I have to sacrifice a lot. "If I were a medical doctor, I would treat all my patients as though they were my own children".

STAGE 10: NATURAL ALTRUISM

All creatures in Nature are lovely and equal. I love humans, animals, and other living things. I love them all. I am willing to sacrifice my life not only for humans but also for other animals. I would also be dedicated to educate people to protect the natural environment for all the living things in Nature. I am part of Nature and I want to live happily and peacefully with all the creatures in Nature. I can't bear to see any damage and disruption to the Nature especially any harm to the life of the wonderful creatures. I would be heart-broken to see and to hear people slaughter any animals. If I can, I would rather be the one to be slaughtered than to see other animals to be slaughtered. "If I don't go to hell, who else will?"
